# Infectious pathogens may trigger specific allo-HLA reactivity via multiple mechanisms

**DOI:** 10.1007/s00251-017-0989-3

**Published:** 2017-07-17

**Authors:** Lloyd D’Orsogna, Heleen van den Heuvel, Cees van Kooten, Sebastiaan Heidt, Frans H.J. Claas

**Affiliations:** 1 Department of Clinical Immunology and Pathwest, Fiona Stanley Hospital and University of Western Australia, Perth, Australia; 2 Department of Immunohaematology and Blood Transfusion, Leiden University Medical Center, Leiden, The Netherlands; 3 Department of Nephrology, Leiden University Medical Center, Leiden, The Netherlands

**Keywords:** Infection, HLA, Alloreactivity, Heterologous immunity, T cells, B cells

## Abstract

Transplant recipients can be sensitized against allo-HLA antigens by previous transplantation, blood transfusion, or pregnancy. While there is growing awareness that multiple components of the immune system can act as effectors of the alloresponse, the role of infectious pathogen exposure in triggering sensitization and allograft rejection has remained a matter of much debate. Here, we describe that exposure to pathogens may enhance the immune response to allogeneic HLA antigens via different pathways. The potential role of allo-HLA cross-reactivity of virus-specific memory T cells, activation of innate immunity leading to a more efficient induction of the adaptive alloimmune response by antigen-presenting cells, and bystander activation of existing memory B cell activation will be discussed in this review.

## Introduction

A substantial portion of the pre-existing T cell repertoire is already alloreactive even in non-sensitized individuals, with pre-existing memory T cells capable of responding to alloantigens. When transplant immunologists quantified the alloreactive T cell repertoire, the proportion of T cells responding to the graft was described as “inordinate” as the number and proportion of T cells responding to donor HLA antigens was found to be vastly greater than that of responses to pathogen-derived antigens (Lombardi et al. 1990; Macedo et al. 2009; Suchin et al. 2001). The pre-transplant frequency of donor-specific IFNγ-producing lymphocytes does correlate with the risk of post-transplant rejection episodes (Heeger et al. 1999). It has been postulated that this high frequency of memory T cells able to respond to allo-HLA even in non-sensitized individuals could be attributable to cross-reactivity from virus-specific memory T cells. Recent in vitro experiments strongly suggest this may indeed be the case (Amir et al. 2010; D’Orsogna et al. 2011a; D’Orsogna et al. 2011b).

B cell sensitization is revealed by the presence of HLA antibodies which are rarely detected in non-sensitized individuals. However, recent evidence confirms that HLA antibody production can also be associated with infections and vaccination.

The role of the innate immune system in solid organ transplant rejection has not been extensively studied. However, it has become clear that also following pathogen infection activation of innate immunity could contribute significantly to the development of acute and/or chronic rejection and impacts alloimmunity.

In this paper, we review how exposure to infectious agents could be associated with increased risk of MHC-specific allorecognition and poor graft outcome in the solid organ transplant setting. Infectious pathogens might induce alloreactivity directly via molecular mimicry (“heterologous immunity”) or alternatively by providing co-stimulatory factors for bystander activation of alloreactive leukocytes. Taken together, recent evidence supports the hypothesis that infectious pathogens may have significant impact on the rate of solid organ rejection. This is also consistent with the notion that infectious pathogens might be a major obstacle to the induction of successful transplant tolerance.

### Allo-HLA cross-reactivity by virus-specific memory T cells

Direct alloreactivity occurs when recipient T cells directly recognize donor cells expressing mismatched HLA molecules, and is usually associated with acute T cell-mediated rejection. The clinical importance of alloreactive T cells activated following transplantation is well documented in the immediate post-transplant period. However, donor cells do continue to express mismatched MHC molecules which could be recognized by directly alloreactive T cells at any time. Directly reactive memory T cell responses to allogeneic MHC may be associated with acute rejection and chronic allograft nephropathy, and are a potent barrier to transplantation tolerance.

In humans, acute rejection has been associated with varying viral infections, and CMV prophylaxis with oral ganciclovir is associated with improved long-term renal graft outcome (Kliem et al. 2008). CMV disease is also associated with increased risk of GvHD in the bone marrow transplant setting (Cantoni et al. 2010). The fact that cord blood T cells are less able to mediate GvHD than marrow derived T cells because of their naive status also supports the theory that memory T cells generated after pathogen exposure are able to directly elicit alloreactive responses (Byrne et al. 1988; Risdon et al. 1995).

The presence of in vivo pathogen-induced alloreactive T cell memory is a potent barrier to transplantation tolerance in mice. Many strategies have been used to successfully induce transplant tolerance in mice, most of which primarily block co-stimulatory pathways such as CD80/CD86/CD28, CD40/CD154, ICOS/ICOSL, or OX40/OX40L among others. For example, donor-specific transfusion and anti-CD154 antibody readily induce tolerance in pathogen-free mice. However, this protocol fails with peri-operative infection with *Listeria monocytogenes* as the pathogen induces memory T cells which abrogate the induction of transplant tolerance (Wang et al. 2008). Furthermore, Adams et al., in an elegant set of experiments, were able to demonstrate a viral dose effect whereby mice previously exposed to multiple viral infections were refractory to tolerance induction and rejected their allografts, whereas tolerance could be induced in naive mice or single pathogen-exposed mice (Adams et al. 2003b). Taken together, these experiments underline the ability of pathogen infection to have a detrimental influence on graft survival and/or tolerance induction.

#### Human EBV-specific clones are cross-reactive against allo-HLA-B*44:02 via molecular mimicry

One potential explanation for the high frequency of alloreactive T cells in non-sensitized individuals is the ability of pre-existing virus-specific T cells to cross-react with allogeneic HLA molecules, a phenomenon termed molecular mimicry or heterologous immunity. To investigate the ability of virus-specific T cells to exert allo-HLA reactivity, virus-specific T cell lines or clones have been tested against panels of donor cells expressing HLA class I and II molecules. EBV EBNA3A-specific T cell clones which are selected to recognize the immunodominant peptide FLRGRAYGL presented on HLA-B*08:01 also recognize allogeneic HLA-B*44:02 and HLA-B*44:05 to which the individual has never been exposed (Burrows et al. 1994; D’Orsogna et al. 2009; Macdonald et al. 2009). Despite extensive polymorphism between HLA-B*08:01, HLA-B*44:02, and HLA-B*44:05 and the disparate repertoire of both viral and allo-peptides, the Epstein-Barr virus (EBV) EBNA3A-specific T cell receptor (TCR, generated against the B*08:01-restricted EBV epitope FLRGRAYGL) engages both B*44:02 or B*44:05 allotypes presenting the self-peptide EEYLQAFTY (from ABCD3 gene) identically, demonstrating intricate mimicry between the peptide-HLA (pHLA) complexes (Archbold et al. 2006; Macdonald et al. 2009). Therefore, virus-specific memory T cells can break the law of HLA restriction and directly recognize foreign HLA molecules from unrelated (allogeneic) individuals (Amir et al. 2010; Archbold et al. 2006; D’Orsogna et al. 2009; D’Orsogna et al. 2010; D’Orsogna et al. 2011a; Macdonald et al. 2009).

#### Allo-HLA reactivity by virus-specific memory T cells is common

The high frequency of allogeneic HLA (allo-HLA) cross-reactivity by virus-specific memory T cells has been confirmed by our group and others (Amir et al. 2010; Burrows et al. 1994; D’Orsogna et al. 2009; D’Orsogna et al. 2010; Macdonald et al. 2009; Rist et al. 2009; Umetsu et al. 1985). Specific allo-HLA cross-reactivity has been shown for EBV, cytomegalovirus (CMV), varicella zoster virus (VZV), and influenza A virus-specific T cells, and the cross-reactivity is mediated by the same T cell receptor (TCR) (Amir et al. 2010; D’Orsogna et al. 2010; D’Orsogna et al. 2012; D’Orsogna et al. 2011a). For example, a CMV pp50/HLA-A1-restricted T cell clone with TCR Vβ3 usage cross-reacts with allogeneic HLA-A*11:01 and a VZV IE62/HLA-A2-specific T cell clone with TCR Vβ14 usage cross-reacts with allogeneic HLA-B*55:01 (Amir et al. 2010). Cross-reactivity for HLA class I-restricted T cell clones with allogeneic HLA class II molecules has also been reported (Amir et al. 2010; Rist et al. 2009). It has been shown that 80% of T cell line lines and 45% of virus-specific T cell clones cross-react (in vitro) with at least one allogeneic HLA molecule (Amir et al. 2010). The allo-HLA cross-reactivity of virus-specific CD8^+^ T cells is exquisitely dependent on the combination of viral cognate peptide, the restricting HLA molecule, and the TCR Vβ usage of the T cell. Therefore, molecular mimicry could underpin human T cell alloreactivity.

Despite a growing awareness of the potential ability of virus-specific T cells to mediate alloimmunity, their involvement in clinical human allograft rejection remains to be proven. Nguyen et al. detected a public CMV-specific CD8 T cell clonotype (NLV-HLA-A2 restricted; TCRαβ TRAV3TRAJ31_TRBV12-4TRBJ1-1) with cross-reactivity with allo-HLA-B27, and showed an expansion of the CMV NLV/HLA-A2 cross-reactive cells prior to CMV reactivation in two lung transplant recipients (Nguyen et al. 2014). However, it could not be confirmed whether the expansion of the CMV-specific T cells in association with active CMV disease was associated with clinically definite allo-B27-mismatched graft rejection (Nguyen et al. 2014; Nguyen et al. 2013). Heutinck and colleagues showed that virus-specific CD8 T cells that recognize both the cognate viral epitope and donor cells are transiently present in the circulation of kidney transplant recipients infected with CMV and EBV (Heutinck et al. 2016). For example, in two HLA-B8+ recipients who received an HLA-B*44:02-mismatched graft, EBV EBNA3A FLR/HLA-B8 cells were detectable in the peripheral blood and remained responsive to donor alloantigen for up to 1 year post transplantation. However, the donor-reactive virus-specific T cell levels declined after transplantation. While a possible explanation for these findings is that the virus-specific T cells migrated to the kidney allograft where they could be harmful, the presence of cross-reactive T cells was not associated with an inferior transplant outcome. The clinical relevance of allo-human leukocyte antigen cross-reactivity in mediating alloimmunity has also been reviewed by Rowntree and colleagues (Rowntree et al. 2016). We suggest that further studies examining the in vivo clinical relevance of allo-HLA cross-reactivity by virus-specific T cells in human transplant recipients are necessary.

#### TCR affinity of cross-reactive virus-specific T cells for allo-HLA and the ability to mediate alloimmunity

During development, T cells undergo an instructional process of positive and negative selection in the thymus, by deletion of T cells from the T cell repertoire that express TCRs with either insufficient or too high affinity for self-HLA. As T cells only encounter self-HLA molecules during their thymic education, shaping of the TCR repertoire does not take into account potential cross-reactivity against allo-HLA molecules. Virus-specific memory T cell clonotypes can therefore theoretically cross-react to allo-HLA with broad variation in TCR affinity. Generally, high-affinity TCR-peptide-MHC (pMHC) interactions are associated with more potent T cell activation compared to low-affinity interactions (Bridgeman et al. 2012; Holler and Kranz 2003; Stone et al. 2009). The differential TCR-pMHC binding associated with variation in affinity induces altered phosphorylation patterns in signaling pathways downstream of the TCR (Madrenas et al. 1995; Sloan-Lancaster et al. 1994), resulting in more potent or even qualitatively different effector functions (Auphan-Anezin et al. 2006; Edwards and Evavold 2011; Jenkins et al. 2009; Nel and Slaughter 2002). Accordingly, TCRs generally bind with higher affinity to agonistic compared to antagonistic peptides (Ely et al. 2005; Lyons et al. 1996). Nevertheless, exceptions to this rule have been described (Kersh et al. 1998) and affinity is subjected to thresholds that prevent further improvement of T cell functionality (Tan et al. 2015).

Variation in TCR affinity for allogeneic HLA ligands is thus likely to infer differential allorecognition, thereby affecting the potential for allograft rejection. For example, TCR affinity has been investigated for the human EBV B8/FLR cross-reactivity against allo-HLA-B*44:02. Using surface plasmon resonance (SPR) and tetramer competition, the TCR affinity for the alloepitope was shown to be significantly lower compared to the cognate epitope (Macdonald et al. 2009), and to date, there is no conclusive evidence for allograft rejection by EBV B8/FLR cross-reactive T cells in a clinical setting. On the other hand, studies of cross-reactive T cells in mice have shown that TCR affinity was significantly higher for the alloepitope compared to syngeneic epitopes (Garcia et al. 1997), leading to potent alloreactive responses in mice. It is evident that more cross-reactivity models should be investigated to make any general statements on the strength of TCR affinity towards the alloepitope—but unfortunately, this research is hampered by the fact that TCR affinity studies require comprehensive knowledge of the allopeptide, which is lacking for most human virus-specific TCR cross-reactivities.

**Fig. 1:**
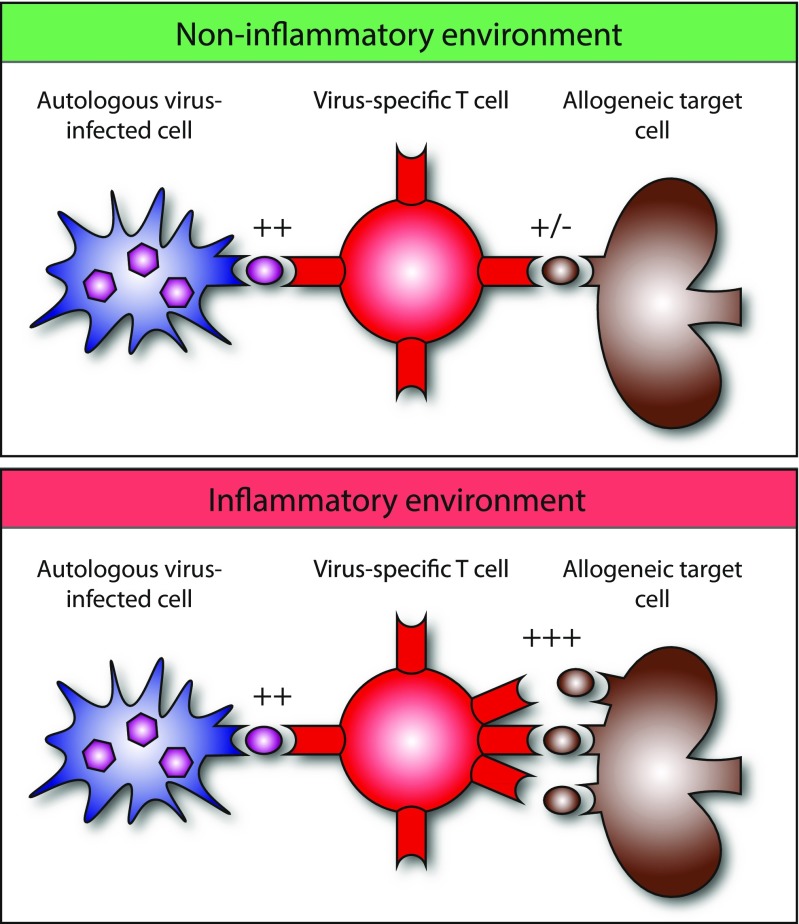
The avidity of virus-induced cross-reactive T cells for a specific HLA alloantigen may depend on the expression of that particular HLA molecule. A higher expression will increase the avidity

Although, in addition to co-stimulation, TCR affinity shows an unmistakable correlation with T cell activation, ultimately the fate and quality of the T cell response is determined by TCR avidity, the accumulated strength of interaction of all non-covalent binding at the T cell surface. The quality of the T cell response is a result of the kinetics of subsequent TCR signaling (Sykulev 2010). Although TCR affinity is considered the most prominent determinant of TCR avidity, other determinants play an important role in contributing to TCR avidity as well: including CD4/CD8 co-receptor binding (Laugel et al. 2007), MHC density on the target cell surface (Corse et al. 2011), cluster formation of TCRs on the T cell surface, the recruitment of signaling molecules to the TCR-CD3 complex, and accessory molecules in lipid rafts (Alonso and Millán 2001). For example, the immunological synapse provides an instrument to amplify signals downstream of lower-affinity TCR interactions, thereby enhancing TCR signaling (Cemerski et al. 2007; Tailor et al. 2008). Furthermore, inflammatory signals lead to the increase of surface MHC expression, thereby in theory increasing TCR avidity for the alloepitope and the likelihood of generating an alloresponse (Fig. 1). On the other hand, for in-depth characterization of TCR-pMHC binding strength, TCR affinity measurement remains the golden standard, determining that TCR avidity under inflammatory and non-inflammatory circumstances may thus provide a biologically relevant surrogate to estimate the binding strength between cross-reactive virus-specific T cells and their allo-HLA target cells when the cross-reactive allopeptide is unknown.

Finally, TCR affinity and avidity determine for a great part TCR signaling required for T cell memory formation (Daniels and Teixeiro 2015). The memory T cell population is of special interest to transplantation, given that it has a lower activation threshold compared to the naive counterpart (Kimachi et al. 2003), the requirement for co-stimulation is abolished, and effector mechanisms are shaped to being most optimal for eradicating virus-infected cells. Indeed, cross-reactive memory T cells have been shown to be able to lead to allograft rejection in mice and pose a threat to tolerance induction (Adams et al. 2003a). Interestingly, a recent report in mice described that even low-affinity priming was able to generate a cross-reactive memory T cell pool that rapidly induced rejection upon high-affinity graft challenge, illustrating the remarkable potential of memory T cells to generate secondary immune responses against cross-reactive epitopes regardless of priming events (Krummey et al. 2016). Increasing the understanding of cross-reactive TCR affinity and avidity for alloepitopes could thus provide better insight into the potential threat of the alloreactive memory T cell compartment under inflammatory and non-inflammatory circumstances.

### Innate immunity

**Fig. 2:**
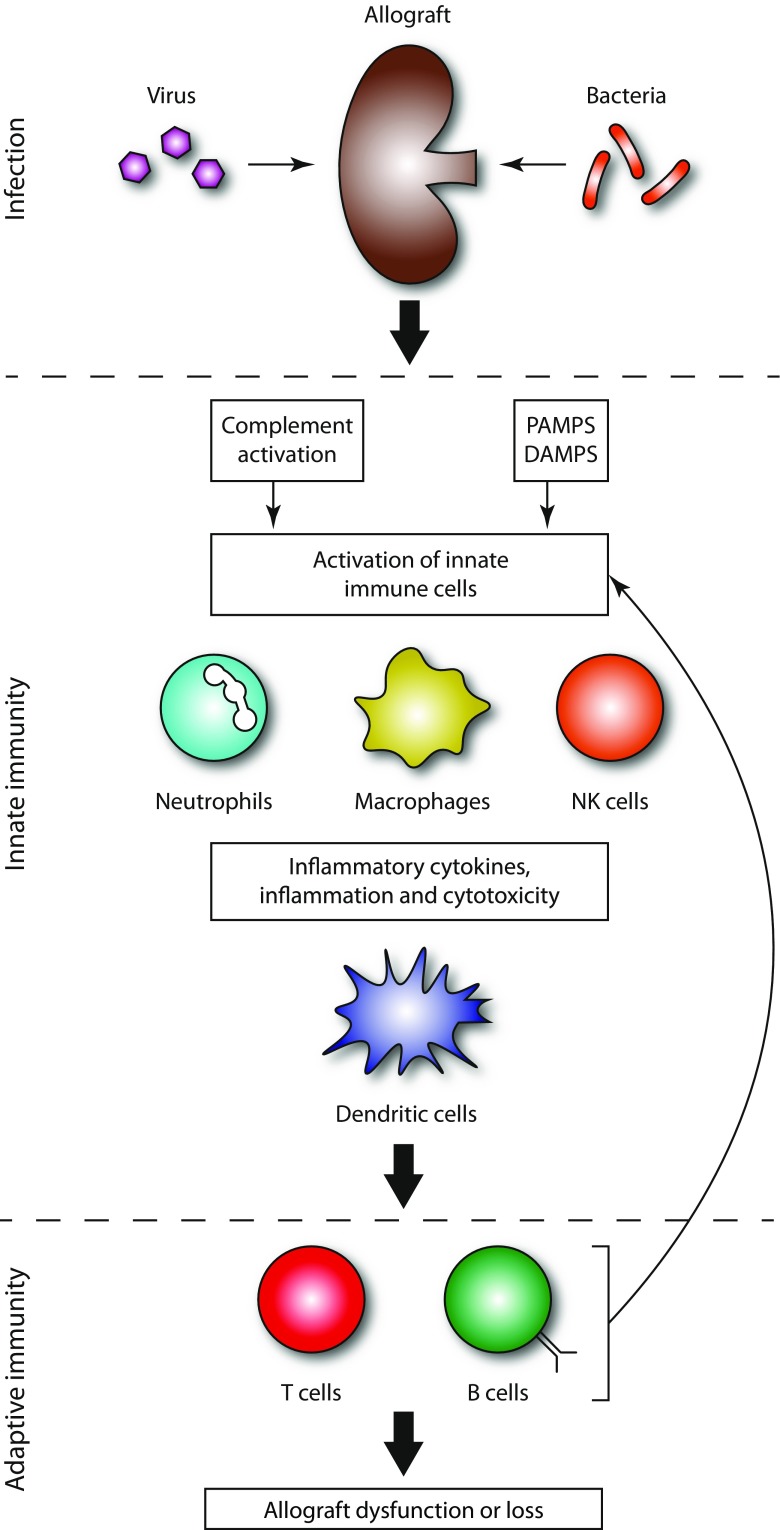
Infectious agents can activate innate immunity via different pathways, which among others may lead to a more efficient presentation of the allogeneic HLA molecules to T cells and B cells

For a long time, solid organ rejection has been considered the consequence of adaptive immunity from cellular and/or antibody-mediated responses. However, there is now consistent evidence that activation of innate immunity is necessary prior to the initiation of allo-HLA-specific immune responses and rejection. The activation of innate immunity following invasion by infectious pathogens could therefore contribute to allorecognition and graft rejection (Fig. 2 **).**

#### Toll-like receptors

Toll-like receptors (TLRs) are pattern recognition receptors that recognize highly conserved pathogen-derived molecules. All TLRs transduce their signal via the activation of the MyD88 protein with the exception of TLR3 which uses the TRIF molecule. TLR1 is ubiquitously expressed and all TLRs are expressed on epithelial cells and TLR5–10 are expressed on endothelial cells and many graft parenchymal cells. TLR activation as a consequence of pathogen infection is associated with potent activation of the innate immune system including secretion of inflammatory cytokines and chemoattractants and maturation and activation of donor and recipient antigen-presenting cells. Therefore, TLRs could be an essential link between innate immunity and adaptive immune responses against alloantigens.

Activation of TLRs following pathogen infection is thought to play a key role in the recruitment and activation of alloreactive lymphocytes associated with graft rejection. There is much support for this link in rodent models. Induced tolerance to cardiac and skin allografts can be overcome by injection of the TLR9 agonist CpG and the TLR2 agonist Pam3CysK (Chen et al. 2006; Porrett et al. 2008; Thornley et al. 2006; Walker et al. 2006). Absence of the MyD88 adaptor protein in both donor and recipient mice has also been associated with acceptance of minor antigen-mismatched grafts (Goldstein 2011; Goldstein et al. 2003; Tesar et al. 2004).

Palmer and colleagues investigated the impact of two functional polymorphisms in the TLR4 gene on the incidence of acute rejection after lung transplantation (Palmer et al. 2003). Patients and donors were screened for the TLR4 Asp299Gly and Thr399Ile polymorphisms, both of which are associated with endotoxin hyporesponsiveness. The rate of acute rejection at 6 months was significantly reduced in recipients with either of these polymorphisms, as compared to the wild type. These results strongly suggest that activation of innate immunity following endotoxin exposure via TLR4 signaling contributes to the development of acute lung transplant graft rejection. More generally, activation of the many different damage-associated molecular pattern (DAMP) receptors and/or pathogen-associated molecular pattern (PAMP) receptors, as a consequence of local or systemic infection, could be associated with increased risk of adaptive immunity and allorecognition. This mechanism may be particularly relevant in lung transplantation given the large pathogen burden and regular exposure to the foreign environment.

#### Pro-inflammatory cytokines and chemokines

Active allograft infection can trigger graft infiltration, activation of the cells of the innate immune system, and secretion of many different pro-inflammatory cytokines. IL-1, IL-6, IL-12, TNF, and IFNα/γ, among many other cytokines and chemokines, can all lead to graft inflammation, activation of adaptive immunity, and cytotoxicity.

Pro-inflammatory cytokines produced following infection can abrogate tolerance induction and even break established tolerance to an allograft in mouse models. Exogenous IL-1 administration at the time of transplant can prevent tolerance induction to skin and islet allografts (Holan 1988; Sandberg et al. 1993). In key experiments, it has been shown that IL1-receptor blockade can impair donor-specific DTH, reduce corneal graft infiltration by antigen-presenting cells, and abrogate second set rejection of skin allografts (Dana et al. 1998; Dana et al. 1997; Dekaris et al. 1999; Yamada et al. 1998; Yamada et al. 1999; Yamada et al. 2000), suggesting that IL1 production is inextricably linked with antigen presentation and adaptive immunity to allografts in mice models.

IL-6 and TNFα enhance pro-inflammatory immunity and render T cells resistant to suppression by Tregs, and their deficiency renders mice susceptible to tolerance induction via co-stimulation blockade (Goldstein 2011; Goldstein et al. 2003; Walker et al. 2006). IL-6 can prevent transplant tolerance to cardiac allografts by promoting the differentiation and activation of CD8+ T cells of the Th17 phenotype (Burrell et al. 2008). Likewise, type 1 interferons have been shown to confer resistance to tolerance. For example, tolerance resistance following *L. monocytogenes* infection, despite co-stimulation blockade, is dependent on production of interferon α and β (Thornley et al. 2007).

GvHD is a complication of allogeneic bone marrow transplantation whereby donor-derived T cells recognize and damage recipient tissue. In the human hematopoietic stem cell transplantation (HSCT) setting, GvHD has been associated with both IL-6 production and also active CMV replication. Hill and colleagues administered the IL-6 inhibitor tocilizimab to HSCT recipients, in addition to standard GvHD prophylaxis, and showed lower rates of acute GvHD (Kennedy et al. 2014). Results presented by this group confirm that an inflammatory cytokine produced following viral infection can lead to adaptive T cell responses against allogeneic HLA. Furthermore, these results suggest that therapeutic inhibition of cytokines or chemokines may be a potential target to prevent HLA-specific T cell alloresponses.

#### Antigen presentation

After phenotypic transition, antigen uptake, and migration to lymphoid tissues, antigen-presenting cells can present alloantigens to immunocompetent cells of the adaptive immune system. Antigen-presenting cells (APCs) that, following antigen uptake, undergo maturation in an inflammatory environment and/or after exposure to pathogen-associated molecular patterns (PAMPs), such as occurs in the presence of active infection, express high levels of HLA class II and co-stimulatory molecules and are potent inducers of alloimmunity (Rogers and Lechler 2001). After APC activation, T cells can recognize and exhibit effector function against allogeneic HLA via direct allorecognition (TCR recognizes intact allogeneic HLA on the surface of the donor cell) or indirect allorecogniton (TCR recognizes peptide fragments from allo-HLA presented on the surface of autologous APCs).

Currently, it is not proven that active infection, like BK virus infection, and subsequent activation of intra-graft antigen-presenting cell function of either donor APCs (direct allorecogniton) or recipient APCs (indirect allorecognition) can definitely trigger de novo allo-HLA-specific T cell-mediated allorecognition. However, APCs represent an essential link between innate and adaptive alloimmunity, and it is likely that APCs activated following infection are able to provide critical co-stimulatory signals and cytokines both at the site of grafting and in the recipient’s lymphoid tissues, and to also serve as APCs for alloantigen presentation to T cells.

Taken together, therapies aimed at inhibiting innate immune activation following infectious pathogens may also represent a novel means to prevent adaptive immunity against allogeneic tissues.

### Viral infections and anamnestic B cell responses

The relationship between viral infections and ensuing HLA antibody production is controversial, and may be dependent on the type of pathogen assessed, whether active infection or vaccination is studied, as well as the definition of a positive result in HLA antibody screenings (Roelen et al. 2012). Many of the reports on the development of HLA antibodies after viral infections are anecdotal, with large controlled and prospective studies on the subject lacking. Regardless, the majority of data in favor of HLA antibody formation upon viral infection or vaccination hint towards activation of pre-existing memory B cells, with an increase in breadth and strength of HLA antibodies as a result. When taking into account the triggers that can lead to memory B cell activation, viral infections certainly have the potential to result in bystander memory B cell activation. First of all, B cells display various TLRs, which as described above can lead to immune cell activation. TLR ligands such as CpG oligodeoxynucleotides (TLR9) and R-848 (Resiquimod, TLR7-8) are often used in the laboratory for polyclonal B cell activation, with the latter being particularly effective at activating memory B cells (Karahan et al. 2014; Pinna et al. 2009). It is therefore conceivable that similar signals could lead to activation of B cells, regardless of their antigen specificity in vivo. Secondly, cytokines produced upon viral infection as described above could lead to B cell activation (Bonig et al. 1998; Vilchez et al. 2002) and work in concert with TLR signaling. Alternatively, there may be heterologous immunity on the level of B cells analogous to T cells, which could result in HLA antibody formation due to epitope similarity with viral antigens. The latter has not yet formally been proven, and will require extensive screening of HLA antibodies towards panels of viral antigens.

There are several reports of elevated plasma cell infiltrates and C4d positivity in biopsies from renal transplant recipients that experience a viral infection coinciding with acute rejection (Aiello et al. 2004; Forman et al. 2004; Khakhar et al. 2003). These plasma cell infiltrates could be due to local differentiation of memory B cells towards plasma cells, as has been shown for chronic rejection as well (Thaunat et al. 2005). A study which systematically addressed the relation between viral infections and HLA antibodies in 35 sensitized renal transplant waitlist patients and 42 patients transplanted after desensitization showed that increases in strength and breadth of HLA antibodies upon viral infection were common (97 and 55%, respectively). The increase in the breadth of HLA antibodies was mainly within the same cross-reactive antigen group (CREG), indicating an expansion of existing specificities without development of new specificities (Locke et al. 2009). Interestingly, a study focusing on the opposite scenario of possible elevation in virus-specific antibody titers upon HLA antibody formation showed that in this situation, the humoral immune response remained HLA specific without an increase in titers of virus-specific antibodies (Krishnan et al. 2013). This might be due to the nature of the activation signals, such as the lack of PAMPS in the setting of allorecognition.

HLA antibody formation upon vaccination has been studied in more detail, albeit no firm conclusions can be drawn (Roddy et al. 2005). When considering influenza vaccination, several studies showed no effect on HLA antibody formation at all (Candon et al. 2009; Kimball et al. 2000), whereas other studies did show a significant percentage of renal transplant recipients developing anamnestic B cell responses and de novo HLA antibodies (Fairhead et al. 2012; Katerinis et al. 2011). The latter report was on a prospective vaccination study including 151 renal transplant recipients of which 15% of patients subsequently developed HLA antibodies, many of them being de novo. Only 12 patients had been immunized previously as determined by HLA antibody positivity at study onset (Katerinis et al. 2011). This indicates either that true de novo HLA antibodies were formed, possibly due to heterologous immunity, or that a previously undetected B cell memory existed in these patients. Indeed, an HLA-specific B cell memory in the absence of detectable serum antibodies has been shown to exist (Karahan et al. 2015a; Snanoudj et al. 2015). Of note, in patients that experienced an increase in the breadth of HLA antibodies, non-DSA was again directed at epitopes shared with donor HLA antigens, although not exclusively (Katerinis et al. 2011). Another study on influenza vaccination in renal transplant recipients reported only de novo HLA antibody formation after vaccination in 12% of patients (Fairhead et al. 2012). Interestingly, all patients that produced de novo HLA antibodies were female, in whom memory B cells due to prior pregnancies may have been present.

**Fig. 3:**
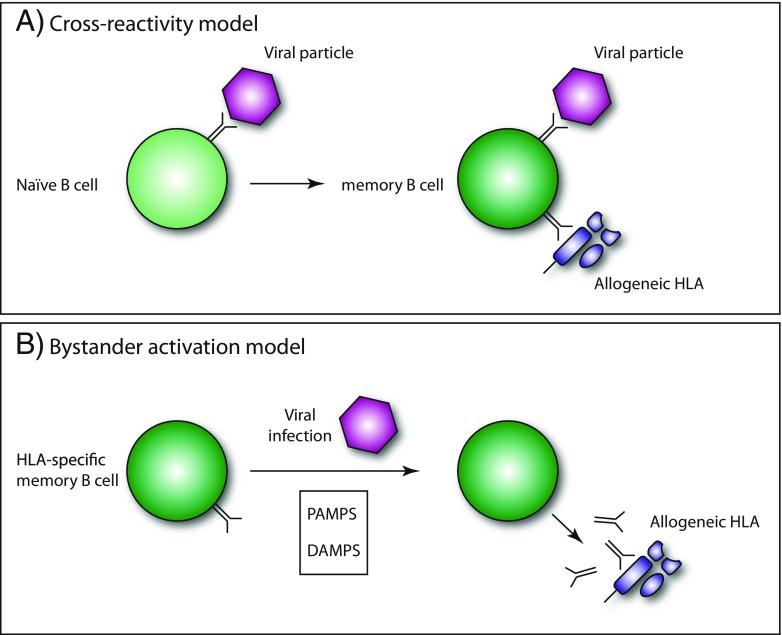
Infectious agents may affect the humoral immune response to allogeneic HLA either by inducing cross-reactive antibodies or by triggering an anamnestic memory B cell response

When considering all data published, it is clear that infection or vaccination may lead to anamnestic memory B cell responses (Fig. 3). It is pivotal to determine what these circumstances are and how memory B cell activation can be prevented. Novel tools to monitor HLA-specific memory B cells will certainly allow to do so (Han et al. 2009; Heidt et al. 2012; Karahan et al. 2015b).

## Conclusions

Taken together, recent scientific evidence supports the hypothesis that infectious pathogens may have a significant impact on the rate of solid organ rejection, and are likely to be a major barrier to successful transplant tolerance, via multiple immunological mechanisms (Amir et al. 2010; Benichou et al. 2012).

It is now firmly established that innate immunity responses triggered after transplantation, as a consequence of tissue damage and infections, can be an essential element of the inflammatory process leading to graft rejection. This review supports the view that activation of the various components of the innate immune system can lead to activation and recruitment of adaptive immunity and transplant rejection. This process can be mediated by TLRs, cytokines, chemokines, and complement and/or antigen-presenting cells. Taken together, these results suggest that tolerance induction protocols will require agents capable of specifically suppressing innate immune responses that are associated with allorecognition, while at the same time not suppressing components of the innate immune system, such as DCs, that are required for transplant tolerance induction (Benichou et al. 2012).

It is undoubted that virus-specific memory T cells are able to exert in vitro allo-HLA-specific reactivity; however, in vivo functional activity of virus-specific memory T cells against allo-HLA is lacking and should be a major focus for investigation. Memory T cells are long-lived, broadly distributed, capable of homing to areas of inflammation, and are rapidly activated after stimulation to exert potent effector function and do remain in the tissue as resident memory lymphocytes. Recent studies have established resident memory T cells as the dominant lymphocyte population surveying most non-lymphoid tissues such as organs. Therefore, their rapid effector function, lower activation requirements, and tissue location suggest that pathogen-specific memory T cells may be a principle mediator of acute and chronic allograft rejection (Beura et al. 2016).

The role of pathogen-specific T cell tissue migration and residency has not been extensively studied in solid organ transplantation but should also become a major focus of investigation. Novel techniques that now allow the tracking of donor-reactive memory T cells may finally be able to determine the clinical relevance of pathogen-specific T cells to allorecognition in the solid organ transplant setting (Beura et al. 2016; Krams et al. 2016; Morris et al. 2015). Accumulating evidence suggests that memory T cells have survival advantages over their counterparts and are more resistant to immunosuppressive medications and lymphoablation, and therefore, if pathogen-specific memory T cells are indeed able to mediate alloreactivity then selective therapies to inhibit alloreactive memory T cells are required (Nicosia and Valujskikh 2016; Valujskikh et al. 2010; Valujskikh and Li 2007). The implications of resident memory T cells for transplantation have been extensively reviewed by Beura et al. (2016).

It is likely that infections and vaccinations can induce anamnestic B cell responses in previously sensitized individuals; however, the ability for infections to induce de novo allo-HLA-specific B cells and antibodies is still uncertain but does require further investigation. New tools developed to monitor HLA-specific B cell responses will provide new insights into the impact of pathogen exposure on the alloreactive B cell repertoire and should help answer some of these important questions (Han et al. 2009; Heidt et al. 2012; Karahan et al. 2015b; Karahan et al. 2014).

Therefore, we argue that infections and vaccinations can stimulate anti-graft responses via multiple mechanisms and could be a major barrier to transplant tolerance. The in vivo relevance of infection and vaccination to allo-HLA-specific reactivity should be a major focus for investigation, and could have major therapeutic implications for treatment of solid organ rejection and induction of transplant tolerance.
